# Multicenter External Validation of the Deep Pelvic Endometriosis Index Magnetic Resonance Imaging Score

**DOI:** 10.1001/jamanetworkopen.2023.11686

**Published:** 2023-05-04

**Authors:** Isabelle Thomassin-Naggara, Michele Monroc, Benoit Chauveau, Arnaud Fauconnier, Pauline Verpillat, Yohann Dabi, Marie Gavrel, Pierre-Adrien Bolze, Emile Darai, Cyril Touboul, Samia Lamrabet, Pierre Collinet, Elise Zareski, Nicolas Bourdel, Horace Roman, Pascal Rousset

**Affiliations:** 1 Department of Radiology, Assistance Publique Hôpitaux de Paris–Hôpital Tenon, Paris, France; 2 Service Imageries Radiologiques et Interventionnelles Spécialisées Sorbonne Université, Paris, France; 3 Department of Radiology, Clinique Saint-Antoine, Bois-Guillaume, France; 4 Radiology Department, CHU Estaing Clermont-Ferrand, Clermont-Ferrand, France; 5 Université Paris-Saclay, UVSQ, Unité de recherche 7285 Risques cliniques et sécurité en santé des femmes et en santé périnatale, Montigny-le-Bretonneux, France; 6 Centre Hospitalier Intercommunal de Poissy-Saint-Germain-en-Laye, Service de Gynecologie et Obstétrique, Poissy CEDEX, France; 7 Department of Radiology, University of Lille, Lille, France; 8 Department of Gynaecology and Obstetrics, Assistance Publique Hôpitaux de Paris–Sorbonne Université, Hôpital Tenon, Paris, France; 9 Department of Radiology, Hospices Civils de Lyon, Lyon Sud University Hospital, Lyon 1 Claude Bernard University, EMR 3738, Pierre Bénite, France; 10 Department of Gynecological and Oncological Surgery, Obstetrics, Hospices Civils de Lyon, Lyon Sud University Hospital, Lyon 1 Claude Bernard University, EMR 3738 CICLY, Pierre Bénite, France; 11 Department of Gynaecology and Obstetrics, Assistance Publique Hôpitaux de Paris–Sorbonne Université, Hôpital Tenon, Paris, France; 12 Department of radiology. Centre Hospitalier intercommunal de Creteil; 13 Hôpital privé Le Bois, Ramsay Lille métropole, Lille, France; 14 Centre Hospitalier Intercommunal de Poissy-Saint-Germain-en-Laye, Service de Radiologie, Poissy CEDEX, France; 15 Gynecology Department, CHU Estaing Clermont-Ferrand, Clermont-Ferrand, France; 16 IFEMEndo, Clinique Tivoli-Ducos, Bordeaux, France

## Abstract

**Importance:**

Preoperative mapping of deep pelvic endometriosis (DPE) is crucial as surgery can be complex and the quality of preoperative information is key.

**Objective:**

To evaluate the Deep Pelvic Endometriosis Index (dPEI) magnetic resonance imaging (MRI) score in a multicenter cohort.

**Design, Setting, and Participants:**

In this cohort study, the surgical databases of 7 French referral centers were retrospectively queried for women who underwent surgery and preoperative MRI for DPE between January 1, 2019, and December 31, 2020. Data were analyzed in October 2022.

**Intervention:**

Magnetic resonance imaging scans were reviewed using a dedicated lexicon and classified according to the dPEI score.

**Main outcomes and measures:**

Operating time, hospital stay, Clavien-Dindo–graded postoperative complications, and presence of de novo voiding dysfunction.

**Results:**

The final cohort consisted of 605 women (mean age, 33.3; 95% CI, 32.7-33.8 years). A mild dPEI score was reported in 61.2% (370) of the women, moderate in 25.8% (156), and severe in 13.1% (79). Central endometriosis was described in 93.2% (564) of the women and lateral endometriosis in 31.2% (189). Lateral endometriosis was more frequent in severe (98.7%) vs moderate (48.7%) disease and in moderate vs mild (6.7%) disease according to the dPEI (*P* < .001). Median operating time (211 minutes) and hospital stay (6 days) were longer in severe DPE than in moderate DPE (operating time, 150 minutes; hospital stay 4 days; *P* < .001), and in moderate than in mild DPE (operating time; 110 minutes; hospital stay, 3 days; *P* < .001). Patients with severe disease were 3.6 times more likely to experience severe complications than patients with mild or moderate disease (odds ratio [OR], 3.6; 95% CI, 1.4-8.9; *P* = .004). They were also more likely to experience postoperative voiding dysfunction (OR, 3.5; 95% CI, 1.6-7.6; *P* = .001). Interobserver agreement between senior and junior readers was good (κ = 0.76; 95% CI, 0.65-0.86).

**Conclusions and Relevance:**

The findings of this study suggest the ability of the dPEI to predict operating time, hospital stay, postoperative complications, and de novo postoperative voiding dysfunction in a multicenter cohort. The dPEI may help clinicians to better anticipate the extent of DPE and improve clinical management and patient counseling.

## Key Points

**Question:**

Does the Deep Pelvic Endometriosis Index (dPEI) magnetic resonance imaging (MRI) score accurately predict operation time, hospital stay, and postoperative complications in a cohort of women undergoing surgery for deep pelvic endometriosis (DPE)?

**Findings:**

In this multicenter cohort study that included 605 adults, operating time and hospital stay were significantly longer for women with severe DPE compared with moderate DPE and for moderate DPE compared with mild DPE. Patients with severe disease were 3.6 times more likely to experience severe complications than those with mild or moderate disease, and lateral locations were associated with a higher likelihood of complications.

**Meaning:**

The findings of this study suggest that clinical application of the dPEI MRI score may assist all clinicians involved in the decision-making process, enabling surgeons to fully inform patients and prepare for the surgical procedure in an optimal manner.

## Teaser

This cohort study examines the use of the Deep Pelvic Endometriosis Index in staging deep pelvic endometriosis based on magnetic resonance imaging findings.

## Introduction

Endometriosis is a frequent gynecologic disorder responsible for severe chronic pelvic pain and infertility. It may substantially alter a patient’s quality of life and represents a considerable burden on women and society.^1,2^ The recent European Society of Human Reproduction and Embryology guidelines recommend first-line hormonal treatment.^3^ If unsuccessful, surgery is an option, but requires detailed evaluation of lesions to assess the risks of surgery and deliver adequate preoperative information to patients.^4^

Preoperative staging of deep infiltrative endometriosis, also known as deep pelvic endometriosis (DPE), is crucial as the surgery can be complex and carries a major risk of intraoperative and postoperative complications and sequelae. These risks require careful preoperative evaluation, and the patient should be included in the decision-making process.^5^ Surgery is led by a gynecologic surgeon with expertise in endometriosis. Additional experts should join the team as required to coordinate complete excision, which may involve various pelvic viscera, such as the rectum, sigmoid colon, bladder, and ureter. In this context of multidisciplinary surgery, preoperative knowledge of the extent of disease and organ involvement is imperative.^4^ Patients should be informed of the risk of postoperative complications, such as rectovaginal fistula, potentially requiring preventive digestive stoma.

Preoperative staging of DPE is currently based on physical examination, transvaginal ultrasonography (TVUS), and magnetic resonance imaging (MRI).^6,7,8^ As the most sensitive technique, MRI allows exhaustive mapping of all locations, especially lateral ones.^9^ Lateral locations comprise parametrial and pelvic wall involvement and require surgical expertise of ureteral and vascular structures as subsequent nerve injuries can be responsible for postoperative voiding dysfunction.^10^

In this setting, the Deep Pelvic Endometriosis Index (dPEI), the first-of-its-kind MRI score, was published in 2020 to standardize pretherapeutic staging through imaging.^11^ This score is the first to include lateral locations of DPE and proposes a compartment-based approach with a score that stratifies the patient according to disease severity. In a single-center retrospective study, the dPEI was validated with operating time, hospital stay, and postoperative complications. Thus, the objective of this study was to externally evaluate use of the dPEI score in a multicenter cohort of women with DPE in predicting postoperative complications.

## Methods

The ENDOVALIRM study was approved by the Hospices Civils de Lyon Institutional Ethics Committee, which granted a waiver of informed consent because deidentified data were used, and the National Institute of Data Protection. This report follows the Strengthening the Reporting of Observational Studies in Epidemiology (STROBE) reporting guideline for observational studies.

### Population

Surgical databases at 7 French endometriosis centers (IFEMEndo Tivoli, Bordeaux, CHU Clermont Ferrand, APHP Sorbonne Université Hopital Tenon, Hospices Civils de Lyon-Hopital Lyon Sud, CH Poissy Saint Germain, CHU Lille, CH Intercommunal de Creteil) were retrospectively queried to identify women who underwent surgery and preoperative MRI for DPE between January 1, 2019, and December 31, 2020. Data were analyzed in October 2022. Exclusion criteria were age younger than 18 years; menopausal status; pregnancy; history of DPE surgery; missing clinical, surgical, pathologic, or imaging data; and an interval of more than 12 months between MRI and surgery. The following clinical criteria were extracted: gravidity, parity, presurgical hormonal treatment, infertility, and clinical symptoms including dysmenorrhea, dyspareunia, pain on defecation or dyschezia, chronic pelvic pain, catamenial diarrhea, rectal bleeding, and preoperative dysuria.

### MRI Acquisition

Magnetic resonance imaging sequences were acquired at 1.5T or 3T using a phased array pelvic coil. The protocol is detailed in eTable 1 in Supplement 1. All MR images were reviewed on a picture archiving and communication system workstation in each center.

### MR Data Analysis

Seven radiologists (including M.M., B.C., P.V., M.G., S.L., and E.Z.), each with a minimum of 5 years’ experience in pelvic MRI, independently reviewed the MR images, blinded to clinical history, surgical examination, and the histologic results, but with knowledge of the presence of DPE. The readers rated the quality of the MR examination.

**Figure 1.**
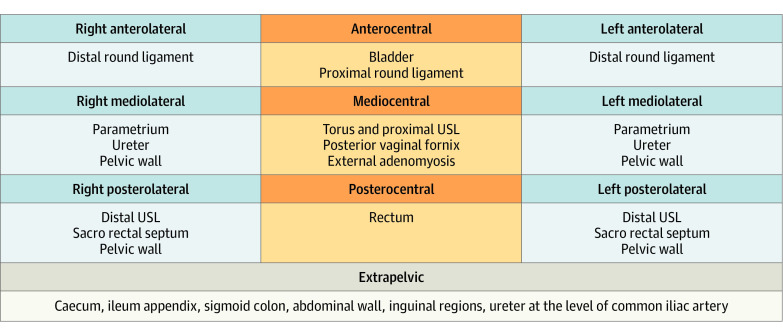
The Deep Pelvic Endometriosis Index (dPEI) Scoring System One point was allocated per compartment where any deep pelvic endometriosis (DPE) lesion was detected and an additional point when an endometriotic lesion involved the pelvic wall in the lateral compartment. One point could be added if a lesion was found in the vagina or in the trigone (defined as the lower part of the bladder base), or if ureteral dilatation was observed. The extent of disease was defined as follows: mild (score ≤2), moderate (scores 3 and 4), and severe (score ≥5). USL indicates uterosacral ligament.

The MR images were subsequently reviewed according to a dedicated lexicon (dPEI)^12^ (eTable 2 in Supplement 1): the readers rated the severity of DPE in each patient according to the dPEI, which divides the pelvis into 9 compartments based on anatomic descriptions.^11,12^ Two horizontal lines divide the pelvis into anterior, median, and posterior parts with an anterior line passing anterior to the cervix or vagina and a posterior line passing anterior to the rectum. Two vertical or ventrodorsal lines divide the pelvis into a central and 2 lateral parts, each line passing from back to front by the uterosacral ligaments (USLs) and the underlying fascia recti, the lateral border of the uterine cervix or vagina, and the lateral wall of the bladder. Extrapelvic disease locations constituted an additional compartment. The DPE lesions were reported as belonging to 1 of the 10 compartments. In the presence of substantial tethering and anatomic distortion due to severe DPE causing projection of a structure into a different compartment, the compartment of the initial location was taken to be that of the involved structure.^12^ In addition, the readers noted the presence of adnexal endometriosis (endometrioma, hematosalpinx, and ovarian implants) and any high T1-weighted (T1W) endometriotic implants in the pelvis. The readers were asked to allocate a dPEI score as published previously (Figure 1).^11,12^

### Reference Standard

The surgical procedures performed included hysterectomy, partial colpectomy, unilateral or bilateral parametrectomy, unilateral or bilateral USL resection, unilateral or bilateral ovarian cystectomy, discoid resection (ie, mechanical resection of the anterior wall of the rectum using an intraluminal circular stapler), segmental digestive resection, rectal shaving, partial bladder resection, unilateral or bilateral ureterolysis, and ureteral reimplantation.^13^ Operating times were also analyzed. The following postoperative data were collected: length of hospital stay; postoperative complications, using the Clavien-Dindo classification during the first postoperative month^14^; presence of de novo voiding dysfunction (defined as a postvoiding residual volume >100 mL at discharge), and late (>1 month) postoperative complications.

### Statistical Analysis

The sample size was based on previous results^11^ and computed to ensure a power of at least 90% (with a 2-sided type I error rate of 5%) to conclude that the dPEI score categories (mild, moderate, and severe) were associated with a different prevalence of complications. Descriptive analysis was performed using the nonparametric Mann-Whitney and Kruskal-Wallis tests for continuous variables. The χ^2^ test was used for categorical or nominal variables. A radiologist not involved in the study read 110 cases from the MRI database to calculate interobserver agreement. Interobserver agreement on the dPEI score categories (mild, moderate, and severe) was assessed by the Cohen κ coefficient.^15^ Bland-Altman plots were used to evaluate concordance between dPEI scores rated by senior and junior readers. Limits of agreement were given as mean (2 SDs), where the mean is ideally 0.^16^

All hypothesis tests were 2-sided, and *P* < .05 was considered to indicate a statistically significant difference. Statistical analyses were performed using MedCalc, version 18.2.1 software (MedCalc Software Ltd).

## Results

### Population

Between January 1, 2019, and December 31, 2020, 1329 women underwent surgery for DPE in the 7 centers. Overall, 724 women were excluded (eFigure 1 in Supplement 1). The final cohort consisted of 605 women with a mean age of 33.3 (95% CI, 32.7-33.8) years.

The population comprised 63.8% (386) nulliparous women including 88.8% (343) who had never been pregnant, 18.5% (112) uniparous women, and 17.7% (107) multiparous women. Overall, 63.6% (385) of the patients were receiving hormonal treatment before surgery. Symptoms, MRI quality, type of surgery, and Clavien-Dindo–graded complications are detailed in Table 1. Mean time between MRI and surgery was 159.3 (95% CI, 151.6-167.1) days. Mean operating time was 152.2 (95% CI, 144.7-159.7) minutes. Mean hospital stay was 3.9 (95% CI, 3.7-4.2) days.

**Table 1.** Characteristics of Women with DPE Undergoing Surgery and Preoperative MRI (N = 605)

Characteristic	No. (%)
Clinical symptom	
Chronic pelvic pain	409 (67.6)
Dysmenorrhea	464 (76.7)
Dyspareunia	372 (61.5)
Defecation pain	244 (40.3)
Infertility	185 (30.6)
Catamenial diarrhea	166 (27.4)
Rectal bleeding	60 (9.9)
Bladder disorders (including dysuria)	185 (30.6)
Abnormal uterine bleeding	72 (11.9)
MRI	
3T	89 (14.7)
Rectal opacification	118 (19.5)
Vaginal opacification	263 (43.5)
Bowel and vaginal opacification	103 (17.0)
Gadolinium injection	79 (13.1)
Thin T2W slices on uterosacral ligaments	331 (54.7)
Favorable subjective rating of MRI quality	558 (92.2)
Type of surgery	
Hysterectomy	106 (17.5)
Partial colpectomy	109 (18.0)
Discoid resection	57 (9.4)
Rectal shaving	130 (21.5)
Colorectal resection	121 (20.0)
Bladder excision	44 (7.3)
Postoperative complication	
Clavien-Dindo grade II	87 (14.4)
Infection	15 (2.5)
Blood transfusion	10 (1.7)
De novo voiding dysfunction	45 (7.4)
Others (eg, pain, ileus)	17 (2.8)
Clavien-Dindo grade IIIA	2 (0.3)
Clavien-Dindo grade IIIB	25 (4.1)
Fistula	9 (1.5)
Hemoperitoneum	9 (1.5)
Abscess	4 (0.6)
Others	3 (0.5)
Clavien-Dindo grade IV	0
Clavien-Dindo grade V	0

Abbreviations: DPE, deep pelvic endometriosis; MRI, magnetic resonance imaging; T2W, T2-weighted.

### MRI Findings

#### Descriptive Analysis

According to the dPEI score, disease was mild in 370 of the 605 women (61.2%), moderate in 156 (25.8%), and severe in 79 (13.1%). Adnexal locations were observed in 218 of 370 women (58.9%) with mild disease, 116 of 156 (74.4%) with moderate disease, and 58 of 79 (73.4%) women with severe disease. Superficial locations were identified in 44 women (11.9%) with mild disease, 19 (12.2%) with moderate disease, and 26 (32.9%) with severe disease. Central endometriosis was described in 564 of the total 605 women (93.2%) and in 329 of 370 (88.9%) with mild disease, all 156 women with moderate disease, and 79 of those with severe disease. Lateral endometriosis was described in 189 women overall (31.2%) and was more frequent in severe disease (78 of 79 [98.7%]) vs moderate disease (76 of 156 [48.7%]) and in moderate disease vs mild disease (25 of 370 [6.7%]; *P* < .001). The prevalence of a DPE lesion in the different compartments is presented in eFigure 2 in Supplement 1. The diagnostic value of the dPEI MRI description related to surgical and pathologic findings is detailed in Table 2. Magnetic resonance imaging accurately predicted the absence of intraoperative findings in all compartments with very low negative likelihood ratios (Table 2).

**Table 2.** Diagnostic Performance of dPEI MRI Description for Predicting Surgical Findings

Compartment	Value (95% CI)					Results, No.			
	Sensitivity	Specificity	Positive likelihood ratio	Negative likelihood ratio	Diagnostic odds ratio	True-positive	True-negative	False-negative	False-positive
Mediocentral	0.89 (0.87-0.91)	NA	0.89 (0.87-0.92)	NA	NA	524	0	60	21
Posterocentral	0.79 (0.74-0.84)	0.77 (0.73-0.81)	3.5 (2.9-4.3)	0.2 (0.1-0.3)	13.6 (9-20.4)	178	296	45	86
Anterocentral^a^	0.73 (0.58-0.84)	0.98 (0.97-0.99)	51.1 (25.2-104.4)	0.27 (0.16-0.43)	189.7 (72.5-496.1)	33	552	12	8
Lateral	0.73 (0.66-0.79)	0.86 (0.83-0.89)	5.5 (4.2-7.2)	0.3 (0.2-0.4)	18.2 (11.8-28.1)	133	368	48	56

Abbreviations: dPEI, Deep Pelvic Endometriosis Index; MRI, magnetic resonance imaging; NA, not applicable.

^a^ Only bladder locations were considered because too many data were missing for surgical location of endometriosis on the proximal round ligament.

#### Association Between dPEI Score and Surgical Outcomes

Operating time was longer for women with DPE in any compartment than for women without DPE. Hospital stay was longer for women with DPE detected in the posterocentral, anterocentral, or extrapelvic compartments than for women without DPE in these compartments (Table 3).

**Table 3.** Diagnostic Performance of dPEI MRI Description for Predicting Surgical Outcomes

Compartment	Presence of DPE locations	Absence of DPE locations in the compartment described	*P* value
Mediocentral^a^			
No. of patients	545	60	NA
Operating time, median (IQR), min	133 (90-100)	88 (56-122)	<.001
Hospital stay, median (IQR), d	48 (16-103)	40 (24-75)	.29
CD grade >II, No. (%)	24 (4.4)	3 (5)	.82
POVD, No. (%)	41 (7.5)	4 (6.6)	.91
Posterocentral^b^			
No. of patients	264	341	NA
Operating time, median (IQR), min	161 (110-241)	106 (73-160)	<.001
Hospital stay, median (IQR), d	93 (27-126)	28 (10-72)	<.001
CD grade >II, No. (%)	19 (7.2)	8 (2.3)	.004
POVD, No. (%)	29 (10.9)	16 (4.7)	.008
Anterocentral^c^			
No. of patients	80	525	NA
Operating time, median (IQR), min	154 (104-245)	120 (80-180)	<.001
Hospital stay, median (IQR), d	76 (24-123)	48 (17-97)	.01
CD grade >II, No. (%)	4 (5.0)	23 (4.3)	.83
POVD, No. (%)	9 (11.2)	36 (6.8)	.02
Mediolaterall^d^			
No. of patients	131	474	NA
Operating time, median (IQR), min	195 (115-207)	118 (80-119)	<.001
Hospital stay, median (IQR), d	51 (7-120)	48 (24-96)	.95
CD grade >II, No. (%)	10 (7.6)	17 (3.5)	.04
POVD, No. (%)	16 (12.2)	29 (6.1)	.01
Posterolateral^e^			
No. of patients	108	497	NA
Operating time, median (IQR), min	150 (90-253)	120 (83-183)	<.01
Hospital stay, median (IQR), d	49 (24-98)	24 (7-101)	.09
CD grade >II, No. (%)	8 (7.4)	19 (3.8)	.10
POVD, No. (%)	16 (14.8)	29 (5.8)	.002
Anterolateral^f^			
No. of patients	19	585	
Operating time, median (IQR), min	109 (90-154)	128 (85-195)	.90
Hospital stay, median (IQR), d	48 (24-112)	48 (22-99)	.26
CD grade >II, No. (%)	0	19 (3.3)	.33
POVD, No. (%)	2 (2.7)	17 (3.2)	.81
Extrapelvic^g^			
No. of patients	96	509	NA
Operating time, median (IQR), min	180 (105-274)	120 (80-182)	<.001
Hospital stay, median (IQR), d	96 (88-139)	48 (16-96)	<.001
CD grade >II, No. (%)	8 (8.3)	19 (3.7)	.04
POVD, No. (%)	6 (6.2)	39 (7.6)	.58

Abbreviations: CD, Clavien-Dindo; DPE, deep pelvic endometriosis; dPEI, Deep Pelvic Endometriosis Index; MRI, magnetic resonance imaging; NA, not applicable; POVD, postoperative voiding dysfunction; USL, uterosacral ligament.

^a^ Torus, proximal USL, posterior vaginal fornix, external adenomyosis.

^b^ Rectum.

^c^ Bladder, proximal round ligament.

^d^ Parametrium, ureteral dilatation, pelvic wall; unilateral or bilateral.

^e^ Distal USL, sacrorectal septum, pelvic wall; unilateral or bilateral.

^f^ Distal round ligament; unilateral or bilateral.

^g^ Cecum, ileum, appendix, sigmoid colon, abdominal wall, inguinal region, ureter at the level of the common iliac artery.

An association was found between the risk of severe (Clavien-Dindo grade>II) postoperative complications and the distribution of DPE in the posterocentral (odds ratio [OR], 3.2; 95% CI, 1.4-7.4; *P* = .004), mediolateral (OR, 2.2; 95% CI, 1-4.9; *P* = .04), and extrapelvic (OR, 2.3; 95% CI, 1-5.5; *P* = .04) compartments. An association was also found between the risk of de novo voiding dysfunction and the involvement of the posterocentral (OR, 2.3; 95% CI, 1.2-4.4; *P* = .008), posterolateral (OR, 2.7; 95% CI, 1.4-5.3; *P* = .002), mediolateral (OR, 2.2; 95% CI, 1.1-4.3; *P* = .01), and anterocentral (OR, 2.3; 95% CI, 1-5.1; *P* = .02) compartments.

#### Association Between dPEI Score and Operating Time, Hospital Stay, and Postoperative Complications

**Figure 2.**
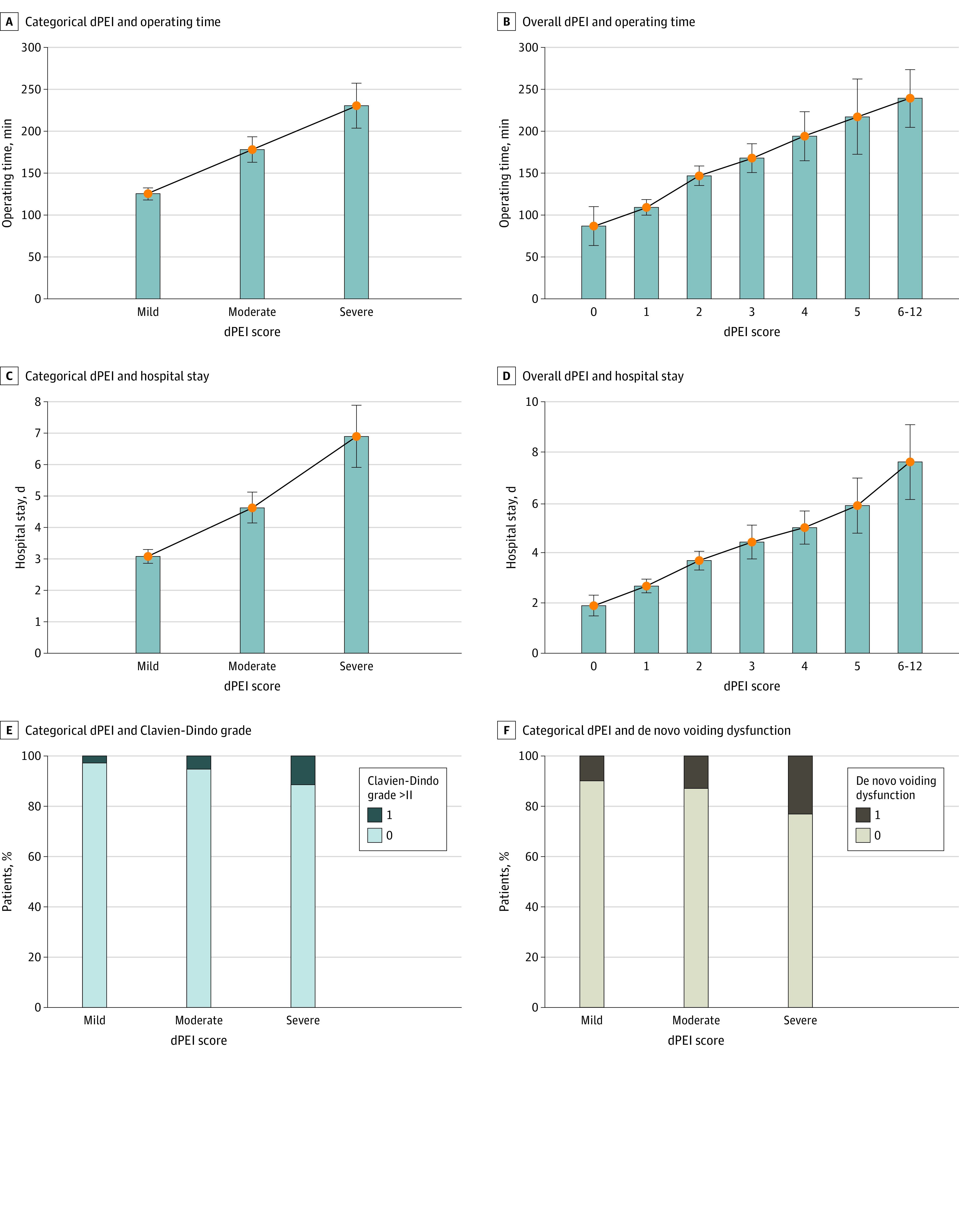
The Deep Pelvic Endometriosis Index (dPEI) Scores and Operating Time, Hospital Stay, Clavien-Dindo Grade, and De Novo Voiding Dysfunction Categorical (A) and overall (B) dePEI and operating time, categorical (C) and overall (D) dPEI and hospital stay, categorical dPEI and Clavien-Dindo grade (E), and categorical dPEI and de novo voiding dysfunction (F). Error bars indicate IQRs.

Operating time differed significantly according to the dPEI score, with longer median times for severe DPE (211 [IQR, 120-330] minutes) than for moderate DPE (150 [IQR, 105-240] minutes) and for moderate DPE than for mild DPE (110 [IQR, 75-165] minutes) (*P* < .001). Moreover, there was a linear increase in operating time according to each point of the score (Figure 2A,B).

Hospital stay also differed according to the dPEI score, with longer median stays for severe DPE (6 [IQR, 4-8] days) than moderate DPE (4 [IQR, 3-6] days) (*P* < .001) and for moderate DPE than mild DPE (3 [IQR, 1-4] days) (*P* < .001). Moreover, there was a linear increase in hospital stay according to each level of the score (Figure 2C,D).

Severe (Clavien-Dindo grade>II) postoperative complications were observed in 10 of 370 patients (2.7%) with mild DPE, 8 of 156 (5.1%) with moderate DPE, and 9 of 79 (11.4%) with severe DPE. Patients with severe disease were 3.6 times more likely to experience severe complications according to the Clavien-Dindo classification than those with mild or moderate disease (OR, 3.6; 95% CI, 1.4-8.9; *P* = .004) (Figure 2E).

Postoperative voiding dysfunction occurred in 19 of 250 (7.6%) women with mild DPE, 13 of 155 (8.4%) with moderate DPE, and 13 of 55 (23.6%) with severe DPE (Figure 2F). Postoperative voiding dysfunction was more frequent in severe DPE than mild or moderate DPE (OR, 3.5; 95% CI, 1.6-7.6; *P* = .001) (eFigure 3 and eFigure 4 in Supplement 1).

### Reproducibility of the dPEI Score

Interobserver agreement was good between senior and junior readers for the 3 categories of the dPEI score (mild, moderate, and severe) (κ = 0.76; 95% CI, 0.65-0.86). When considering the scores as continuous variables, analysis of the Bland-Altman plot (eFigure 5 in Supplement 1) comparing scores between junior and senior readers showed highly accurate concordance, illustrating that the magnitude of the difference does not depend on the severity of the score.

## Discussion

Our results suggest the ability of the dPEI score to predict operating time, hospital stay, postoperative complications, and de novo postoperative voiding dysfunction in a multicenter cohort of women undergoing surgery for DPE. This study noted the importance of identifying lateral locations of DPE, which are mainly involved in high dPEI scores depicting severe disease.

Deep pelvic endometriosis occurs in younger women and is usually diagnosed after a long history of symptoms and therapeutic wandering. The consequent negative outcomes in quality of life are very large.^1,2^ Predicting postoperative complications is crucial. Our study provides external support for the value of the dPEI score based on MRI reporting of different DPE locations. Beyond the goal of diagnosing a disease, the objective of an imaging classification is to standardize interpretation of images. This is important in routine multidisciplinary sessions to discuss treatment options for a patient with endometriosis as various subspecialities are often involved. Furthermore, the dPEI provides a simple communication tool to help patients make an informed decision on which surgical strategy to pursue. Using a simple score, the clinician can accurately inform patients about the risk of complications, which occur in less than 3% of patients with mild disease and more than 10% of those with severe disease. This information may generate a discussion on the benefit-risk ratio and legitimate surgical management. Moreover, when surgery is indicated, the dPEI score offers reliable information about hospital stay (from 3 to 6 days). This places the patient at the center of decision-making and treatment. From a surgical perspective, the dPEI accurately predicts operating times, which range from approximately 2 hours for mild DPE to 3.5 hours for severe DPE. This may help to optimize surgical planning and alleviate economic burden by better predicting operating room occupancy times as well as improving coordination between different surgical subspecialities.

Accurate preoperative assessment should help surgeons anticipate challenging complications and the potential need for preoperative hormonal treatment, such as a gonadotropin-releasing hormone analog.^17^ Predicting the occurrence of de novo voiding dysfunction is of major importance. Although rated as a Clavien-Dindo grade II complication, it is one of the most dreaded complications; it requires self-catheterization and is a major determinant of postoperative alteration of quality of life,^18^ with a risk of definitive sequelae in up to 3% of patients.^19,20,21^ To our knowledge, no studies to date, even those using urodynamic investigation, have been able to predict its occurrence. The dPEI considers the risk of de novo voiding dysfunction by increasing the score if there is involvement of the posterolateral or mediolateral compartments to which the sacral plexus, inferior hypogastric plexus, and splanchnic nerves belong and may be injured.^20^ Thus, the risk of bladder dysfunction is higher in moderate and severe disease. By clearly identifying the lateral compartments, the dPEI provides a practical classification system that is associated with surgical concerns and overcomes the limitations of previous lexica and structured reporting that do not include lateral endometriosis as such.^22,23^

The dPEI score is based on a structured published lexicon with a precise description of each DPE location. This probably explains the good reproducibility of scores between junior and senior readers.^12^ Standardization is critical for the staging of DPE, as there is a wide variety of locations. Moreover, in the 2 main diagnostic techniques—TVUS and MRI—there is a lack of reproducibility for some locations, especially for the USLs, which are the most frequent location.^24^ In this setting, and mainly for TVUS, many imaging classification systems have been published to better stage the disease preoperatively, such as the revised American Society for Reproductive Medicine system, the Endometriosis Fertility Index, and the enzian classification.^25^ However, while TVUS is an appropriate technique for central locations, it is inaccurate for lateral locations, which are mainly involved in the occurrence of postoperative complications and especially voiding dysfunction, as noted in the present study. This is probably why most of these classifications do not include the description of lateral locations. The enzian score is the most widely studied surgical score in MRI.^26^ In the study where the dPEI was first defined, the MRI-based enzian classification was as accurate as but less reproducible than the dPEI due to a low concordance for B lesions (involving the USLs, pelvic wall, and cardinal ligaments) (34.7%).^11^ A recent revised version—#Enzian—is more exhaustive and includes the classification of superficial, ovarian, deep, and extragenital endometriosis and pelvic adhesions.^27,28^ However, #Enzian was built from a surgical perspective and only the radiologist visualizes the whole pelvis in transection. The surgeon visualizes the peritoneum and exterior surfaces of the underlying organs by laparoscopy and thus surgical laparoscopic classifications do not provide an overall evaluation of DPE, particularly in cases of extensive parametrial involvement.^29^ The surgeon cannot initially visualize the insides of organs or subperitoneal or retroperitoneal spaces, or the depth or extent of DPE infiltration into the organs, especially in the case of cul-de-sac obliteration. Most endometriosis surgeons agree that they are only able to find the lesions they are looking for. Thus, preoperative assessment is crucial to guide the dissection intraoperatively.

The dPEI score was mainly designed as a quantitative score taking into account lesion distribution in pelvic quadrants based on the Peritoneal Cancer Index model used for peritoneal metastases.^30^ However, unlike the Peritoneal Cancer Index, which awards 1 to 3 points according to the size of the peritoneal metastasis, the dPEI does not consider lesion size. Given the absence of an established size threshold and the inherent interobserver variability in measuring lesions, we preferred to adopt a complementary semiqualitative approach with minimal modification to the initial version of the dPEI by adding just 1 point for lesions involving more complex surgical procedures and a compartment for extrapelvic lesions.

### Limitations

This study has limitations beyond its retrospective nature, which implies an inevitable risk of bias and missing data. First, the patients included in the study who underwent MRI in the 7 centers were mostly referred by highly experienced tertiary centers and were more likely to have extensive endometriosis, which constitutes a recruitment bias. Second, we had a relatively low number of patients with lateral pelvic wall endometriosis, probably due to the low prevalence of this disease presentation. However, our multicenter study involved 605 patients and is representative of this rare location. Third, long-term follow-up was not performed, with the consequent lack of data about the mean time of intermittent self-catheterization and outcomes on pain relief and fertility rates.

## Conclusion

This study noted the accuracy of the dPEI score in a multicenter cohort and underlines the importance of preoperatively identifying lateral locations of DPE. The dPEI score may assist all clinicians involved in the decision-making process, helping surgeons to fully inform the patient and be able to prepare for the surgical procedure in an optimal manner.

## Notes

Supplement 1. **eTable 1.** Magnetic Resonance Imaging Protocol **eTable 2.** Definitions of Magnetic Resonance Imaging (MRI) Locations According to ENDOVAL IRM Lexicon **eFigure 1.** Flowchart Showing Patient Population and Inclusion Table **eFigure 2.** Prevalence of DPE Locations According to dPEI **eFigure 3.** Mild DPE in a 25-Year-Old Woman With Mediocentral Locations Only **eFigure 4.** Severe DPE in a 39-Year-Old Woman With DPE Locations in 6 Compartments **eFigure 5.** Bland-Altman Plots to Evaluate Concordance Between dPEI Scores by Junior and Senior Readers

Supplement 2.
Data Sharing Statement
