# Supplementary material for: Multicenter External Validation of the Deep Pelvic Endometriosis Index Magnetic Resonance Imaging Score
Source: JAMA Netw Open. 2023 May 4;6(5):e2311686. doi: 10.1001/jamanetworkopen.2023.11686 (PMC10160872; doi:10.1001/jamanetworkopen.2023.11686)
Supplement: Supplement 1. — eTable 1. Magnetic Resonance Imaging Protocol eTable 2. Definitions of Magnetic Resonance Imaging (MRI) Locations According to ENDOVAL IRM Lexicon eFigure 1. Flowchart Showing Patient Population and Inclusion Table eFigure 2. Prevalence of DPE Locations According to dPEI eFigure 3. Mild DPE in a 25-Year-Old Woman With Mediocentral Locations Only eFigure 4. Severe DPE in a 39-Year-Old Woman With DPE Locations in 6 Compartments eFigure 5. Bland-Altman Plots to Evaluate Concordance Between dPEI Scores by Junior and Senior Readers [file jamanetwopen-e2311686-s001.pdf]

Supplementary Online Content

Thomassin-Naggara I, Monroc M, Chauveau B, et al. Multicenter external validation of the deep pelvic endometriosis index magnetic resonance imaging score. *JAMA Netw Open*. 2023;6(5):e2311686. doi:10.1001/jamanetworkopen.2023.11686

eTable 1. Magnetic Resonance Imaging Protocol

eTable 2. Definitions of Magnetic Resonance Imaging (MRI) Locations According to ENDOVAL IRM Lexicon

eFigure 1. Flowchart Showing Patient Population and Inclusion Table

eFigure 2. Prevalence of DPE Locations According to dPEI

eFigure 3. Mild DPE in a 25-Year-Old Woman With Mediocentral Locations Only

eFigure 4. Severe DPE in a 39-Year-Old Woman With DPE Locations in 6 Compartments

eFigure 5. Bland-Altman Plots to Evaluate Concordance Between dPEI Scores by Junior and Senior Readers

This supplementary material has been provided by the authors to give readers additional information about their work.

© 2023 Thomassin-Naggara I et al. *JAMA Network Open.*

eTable 1: Magnetic Resonance Imaging Protocol.

**Minimal protocol**	
Sagittal 2D T2W sequence	Slice thickness ≤ 5 mm<br>Maximal interslice gap 1 mm<br>From one hip to the other
Pelvic axial 2D T2W sequence	Slice thickness ≤ 5 mm<br>Maximal interslice gap 1 mm
2D axial T1W with and without fat saturation	Slice thickness ≤ 3mm<br>No gap
**Optional sequence**	
3D T2W sequence	Isotropic sequence
Axial T2W thin slice in the uterosacral ligament	Slice thickness ≤ 3mm<br>No gap
Coronal T2W sequence or additional axial T2W slices	Evaluation of pyeloureteral dilatation
T1W sequence with gadolinium injection	

© 2023 Thomassin-Naggara I et al. *JAMA Network Open.*

eTable 2. Definitions of Magnetic Resonance Imaging (MRI) Locations According to ENDOVAL IRM Lexicon.[12]

Anatomic structure	MRI reporting
	Left and right Anterolateral compartments
Distal round ligament	Fibrotic unambiguous nodular thickening (generally > 1 cm) +/- hemorrhagic cystic area<br>“Distal” corresponds to the intrapelvic two-thirds of the round ligament, up to the deep orifice and/or into the inguinal canal.
	Anterocentral compartment
Proximal round ligament	Fibrotic unambiguous nodular thickening (generally >1 cm) +/- hemorrhagic cystic area<br>Often associated with external adenomyosis<br>“Proximal” corresponds to intrapelvic third of the round ligament.
Bladder	Nodule or mass involving the muscularis layer<br>3 different locations within the bladder wall:<br>• Anterior dome<br>• Vesicouterine pouch<br>• Bladder base (trigone corresponding to its lower part)
	Mediocentral compartment
Torus and proximal uterosacral ligaments (USLs)	“Proximal” = < 2 cm from the torus<br>Positive diagnosis, with or without hemorrhagic implants, if<br>• regular or irregular fibrotic thickening > 5 mm<br>• nodular (defined as if nodule is present in 2 planes)<br>• retraction (spiculate defined as irregularity present in 2 planes, distortion of shape, surrounding fat infiltration)<br>“Uncertain” diagnosis if<br>• irregular or pseudo nodular (defined as present in only 1 plane)<br>• fibrotic thickening between 3 and 5mm
Posterior vaginal fornix	Nodule or thickening of the posterior vaginal wall.
Rectovaginal septum and anterior mesorectum	Nodule or mass located or extended between the vagina and the rectum, below the peritoneal pouch of Douglas reflection
External adenomyosis (anterior or posterior)	Nodular fibrotic extrinsic infiltration of the myometrium with ill-defined borders, +/- hemorrhagic cystic area.
	Left and right Mediolateral compartments
Parametrium	Irregular and/or retractile fibrotic infiltration +/- hemorrhagic cystic area of vascular and adipocellular tissue of lateral or posterolateral cervical or vaginal regions, between the anterior and posterior lines.
Ureter	Fibrotic infiltration with abolition of the periureteric cellular and fat signal at the entry of the ureter in the parameter or more posteriorly at the level where it ran behind the posterior leaf of the broad ligament<br>+/- ureteral dilatation
Uterine artery	Fibrotic infiltration surrounding the uterine artery (serpiginous flow void) with abolition of the periartery cellular and fat signal
Pelvic wall, external iliac, and/or obturator vessels	Fibrotic infiltration of external iliac and obturator vessels and obturator muscles, up to the pelvic wall/obturator muscle
	Posterocentral compartment
Rectum and rectosigmoid junction	1) Thickening or mass of the rectosigmoid wall<br>2) Location within the rectosigmoid, considering the distance between the anal verge and the middle point of the rectal lesion, as follows:<br>• Lower rectum (0-5 cm)

© 2023 Thomassin-Naggara I et al. *JAMA Network Open.*

	• Middle rectum (5-10 cm)<br>• Upper rectum (10-15 cm)<br>• Rectosigmoid junction (>15 cm)
**Posterolateral compartments**	
Distal USL	"Proximal" = internal one-third of USL (< 2 cm from the torus)<br>Positive diagnosis, with or without hemorrhagic implants, if<br>• regular or irregular fibrotic thickening >5 mm<br>• nodular (defined as if nodule is present in 2 planes)<br>• retraction (spiculate defined as irregularity present in 2 planes, distortion of shape, surrounding fat infiltration)<br>"Uncertain" diagnosis if<br>• irregular or pseudonodular (defined as present in only 1 plane)<br>• fibrotic thickening between 3 and 5 mm
Sacro-recto-genital septum	Sacro-recto-genital septum involvement, either unilateral or bilateral, reported on T2-weighted images in the presence of the loss of the normal aspect of the subperitoneal pararectal cellular sheet, under the uterosacral ligament, with a hypointense thickening of the fascia recti extending posteriorly from the rectovaginal septum
Sacral roots, sciatic nerve, internal iliac vessels, and pelvic wall	Fibrotic infiltration of sacral roots, sciatic nerve and internal iliac vessels, and pelvic wall, often in continuity with infiltration of the sacro-rectal septum.
**Extra-pelvic locations**	
Caecum-ileum-appendix	Reporting of each lesion independently.<br>Fibrotic nodular infiltration of the cecum and ileum (+/- ileum distension) including distance to the ileocecal valve or appendicular wall (+/- appendix distension)
Sigmoid colon	Lesion located above the rectosigmoid junction, indicating the proximal or distal part of the sigmoid
Abdominal wall	Nodule +/- hemorrhagic cystic area of the rectus muscles and/or lateral muscles<br>Umbilical nodule
Inguinal regions	Nodular fibrotic infiltration +/- hemorrhagic cystic area of the terminal extrapelvic part of the round ligament at the level of the superficial orifice of inguinal canal or in the labia majora.
Ureters at the level of common iliac artery	Fibrotic infiltration with abolition of the peri ureteric fat signal+/- ureteral dilatation.

© 2023 Thomassin-Naggara I et al. *JAMA Network Open.*

eFigure 1 Flowchart showing patient population and inclusion table

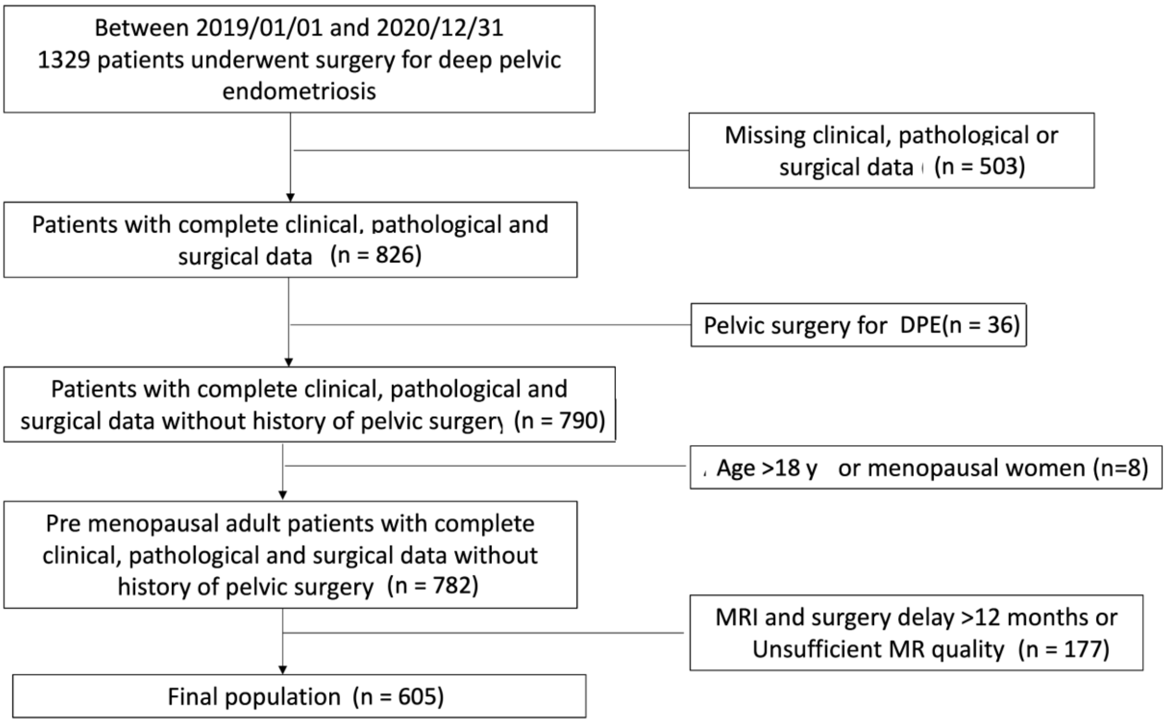

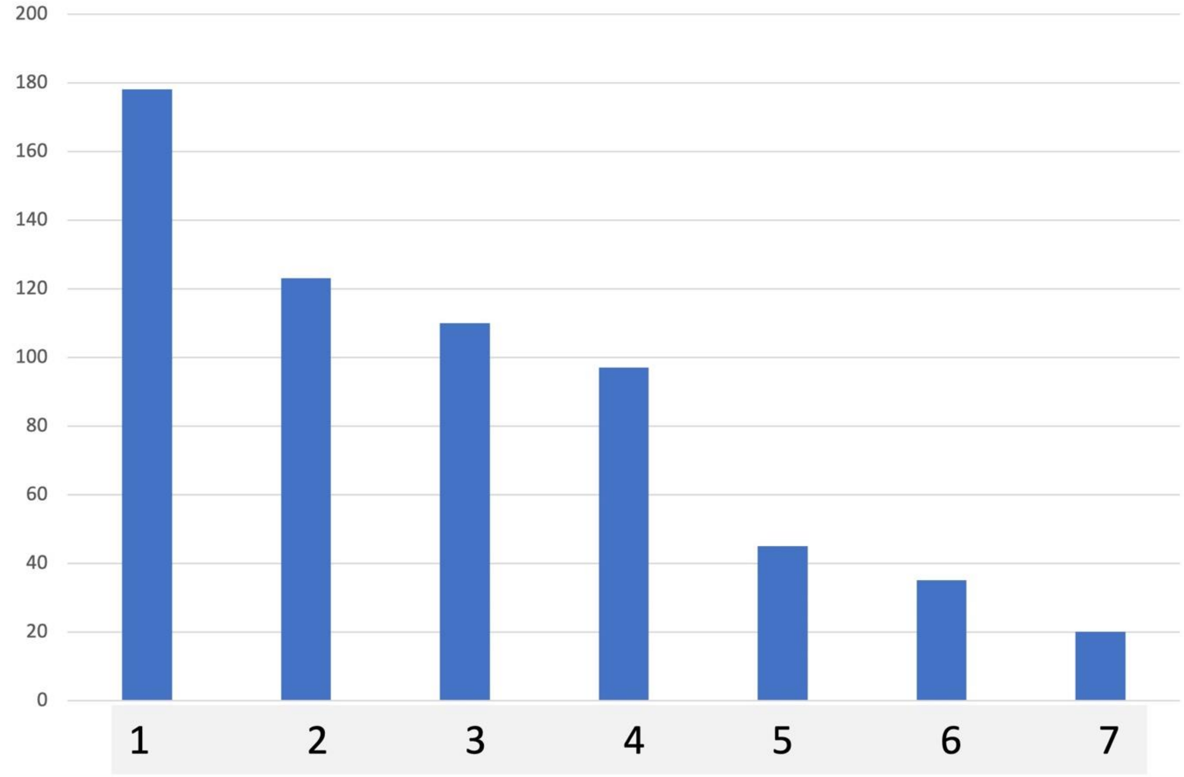

1, IFEMEndo Tivoli, Bordeaux; 2, CHU Estaing Clermont-Ferrand ; 3, Hôpital Tenon; 4, Hospices Civils de Lyon-CHU Lyon Sud; 5, Centre Hospitalier Intercommunal de Poissy-Saint-Germain-en-Laye ; 6, CHU de Lille; 7, Centre Intercommunal de Creteil.

© 2023 Thomassin-Naggara I et al. *JAMA Network Open.*

eFigure 2: Prevalence of DPE Locations According to dPEI.

**Right anterolateral**<br>**N=12 (2%)**<br>- **Distal round ligament** (n = 12/2%)	**Anterocentral**<br>N = 80 (13.2%)<br>- **Trigone of the bladder**[a] (n = 14/2.3%)<br>- **Vesico Uterine Pouch** (n = 27/4.4%)<br>- **Dome of the bladder** (n = 11/1.8%)<br>- **Proximal round ligaments** (n = 47/7.7%)	**Left anterolateral**<br>N = 8 (1.3%)<br>- **Distal round ligament** (n = 8/1.3%)
**Right mediolateral**<br>N = 86 (14.2%)<br>- **Parametrium** (n = 86/14.2%)<br>- **Ureteral dilatation**[a] (n = 18/2.9%)<br>- **Pelvic wall**[a] (n = 6/0.8%)	**Mediocentral**<br>N = 545 (90.1%)<br>- **Torus** (n = 436/72%)<br>- **Right Proximal USL** (n = 395/65.2%)<br>- **Left Proximal USL** (n = 362/59.8%)<br>- **Posterior vaginal fornix**[a] (n = 153/25.3%)<br>- **External adenomyosis** (n = 177 / 29.1%)	**Left mediolateral**<br>N = 82 (13.6%)<br>- **Parametrium (**n = 82/13.6%)<br>- **Ureteral dilatation**[a] (n = 11/1.8%))<br>- **Pelvic wall**[a] (n = 5/0.8%)
**Right posterolateral**<br>N = 67 (11.1%)<br>- **Distal USL** (n = 56/9.2%)<br>- **Sacro-recto-genital septum**<br>(n = 33/5.4%)<br>- **Pelvic wall**[a] (n = 10/1.6%)	**Posterocentral**<br>N = 264 (43.6%)<br>- **Low rectum** (n = 14/5.3%)<br>- **Mid rectum** (n = 101/16.6%)<br>- **High rectum** (n = 149/24.6%)	**Left posterolateral**<br>N = 63 (10.4%)<br>- **Distal USL** (n = 5/8.2%)<br>- **Sacro-recto-genital septum**<br>(n = 43/7.1%)<br>- **Pelvic wall**[a] (n = 11/1.8%)
	**Extrapelvic**<br>N=96 (15.9%)<br>- **Caecum, ileum, appendix** (n = 14/2.3%)<br>- **Sigmoid colon** (n = 62/10.2%)<br>- **Abdominal wall** (n = 10/1.6%)<br>- **Inguinal regions** (n = 4/0.6%)<br>- **Ureter at the level of common iliac artery**<br>(n = 9/1.5%)	

[a] When this location was described, an additional point was added to the final score.

© 2023 Thomassin-Naggara I et al. *JAMA Network Open.*

eFigure 3: Mild DPE in a 25-year-old woman with mediocentral locations only (torus + proximal uterosacral + posterior vaginal pouch). Assigned a dPEI score of 2 (1 compartment + 1 point for vaginal location). Operating time was 98 minutes, hospital stay was 2 days, and no postoperative complications were recorded. DPE indicates deep pelvic endometriosis; dPEI, deep Pelvic Endrometriosis Index.

A Sagittal TSE T2 showing thickened posterior vaginal pouch (arrow)

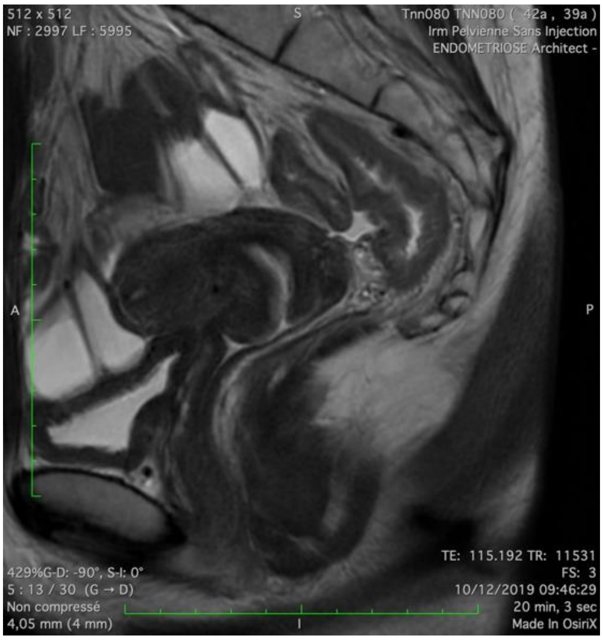

B Axial EG T1 with FAT SAT showing hemorragic implant located in posterior vaginal pouch (arrow).

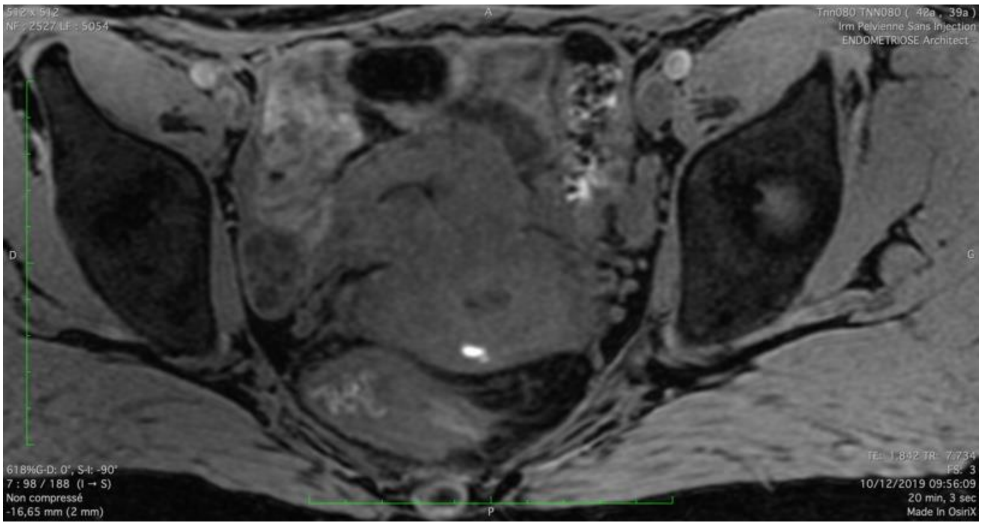

© 2023 Thomassin-Naggara I et al. *JAMA Network Open.*

C Axial oblique TSE T2 3mm showing smooth thickened torus uterinum and left proximal uterosacral ligament (arrow).

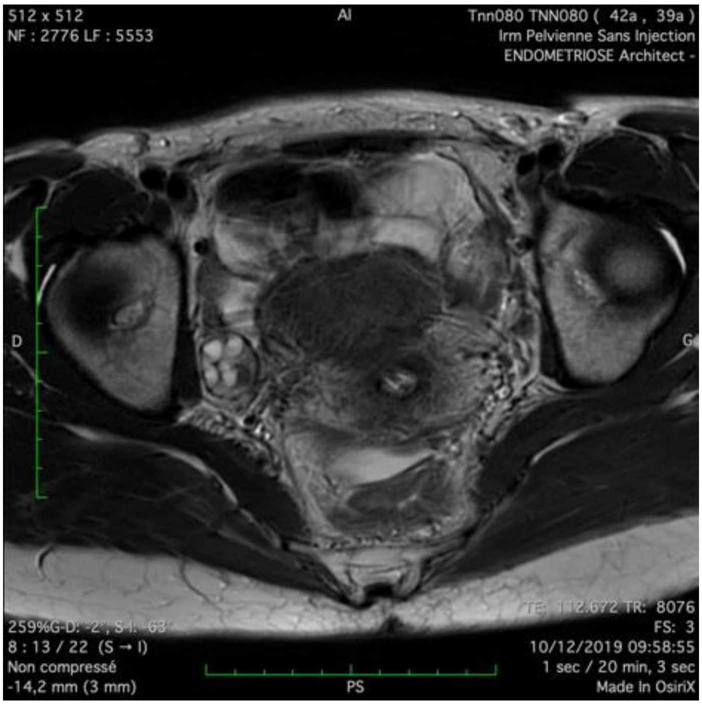

D Axial EG T1 with FAT SAT showing hemorragic implant located in left proximal uterosacral ligament (arrow).

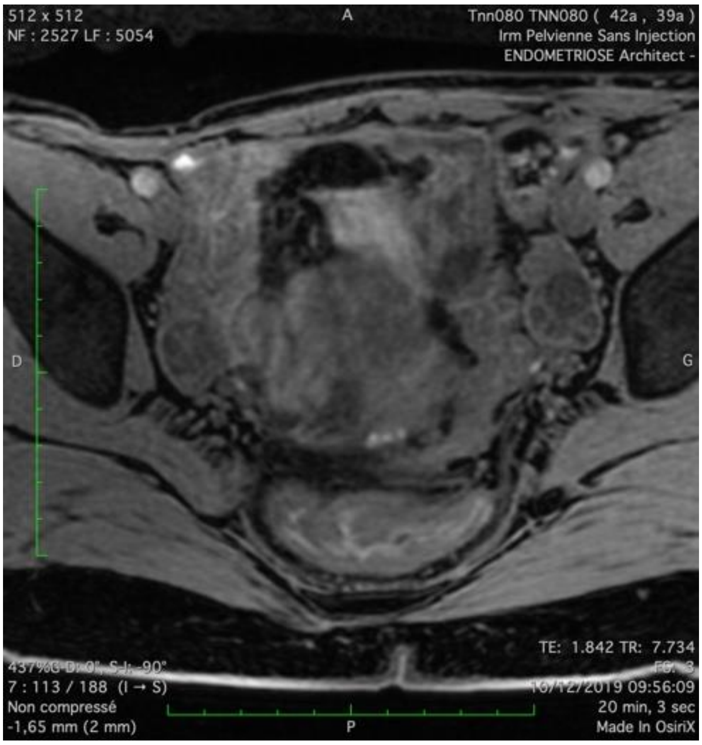

© 2023 Thomassin-Naggara I et al. *JAMA Network Open.*

eFigure 4: Severe DPE in a 39-year-old woman with DPE locations in 6 compartments including bilateral lateral locations with involvement of right mediolateral compartment (due to right parametrial location) and left posterolateral compartiment (due to left sacrorectogenital septum). Assigned a dPEI score of 5. Operating time was 209 minutes, hospital stay was 12 days, and the patient experienced *de novo* voiding dysfunction up to 1 month after surgery. DPE indicates deep pelvic endometriosis; dPEI, deep Pelvic Endrometriosis Index.

A Axial oblique TSE T2 3mm in the plane of the uterosacral ligaments showing nodular thickening of the torus uterinum, a severe deep infiltrating endometriosis lesion of the right parametrium (arrow) without ureter involvement and deep infiltrating endometriosis of the left sacrorectal septum without extension to the pelvic wall.

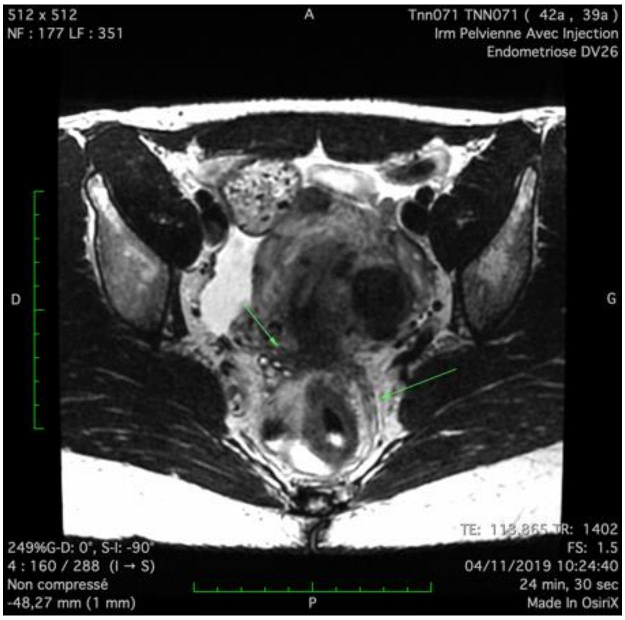

B Right parasagittal TSE T2 showing parametrial location.

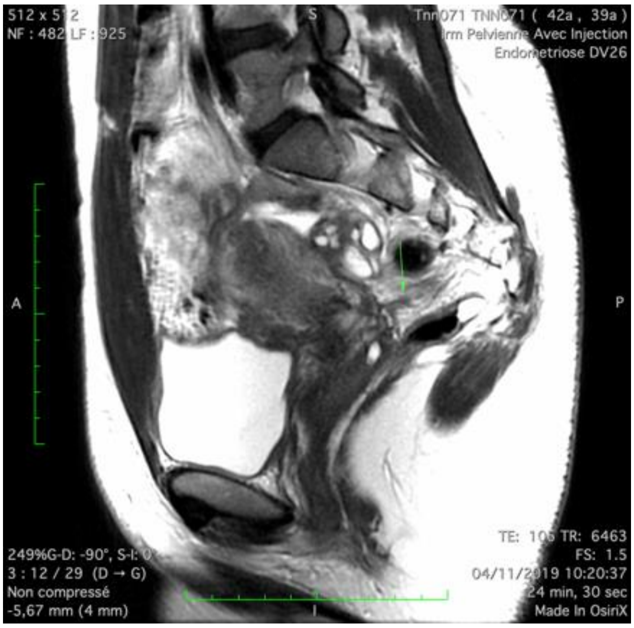

C Left parasaggital TSE T2 showing upper involvement of sacrorectal septum.

© 2023 Thomassin-Naggara I et al. *JAMA Network Open.*

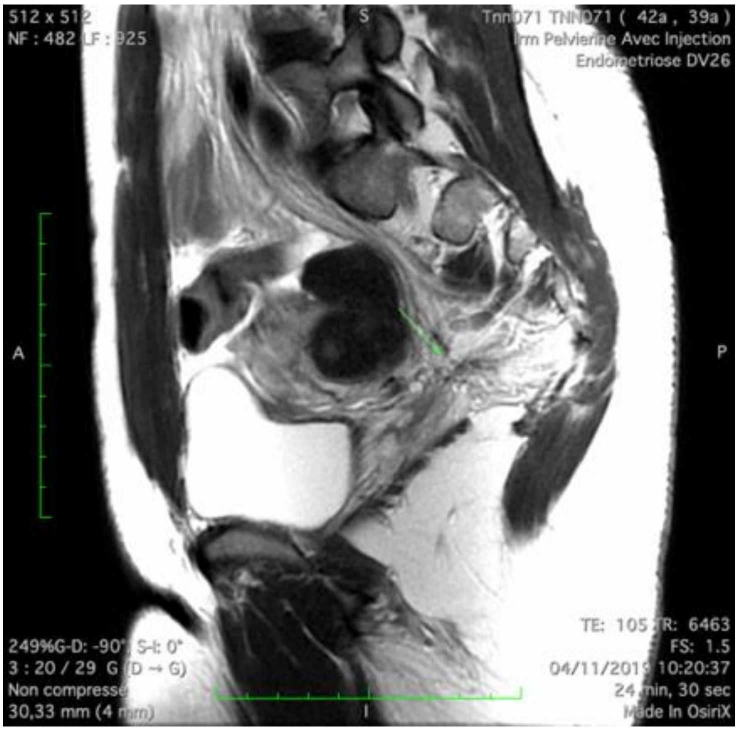

© 2023 Thomassin-Naggara I et al. *JAMA Network Open.*

eFigure 5: Bland-Altman Plots to Evaluate Concordance Between dPEI Scores by Junior and Senior Readers.

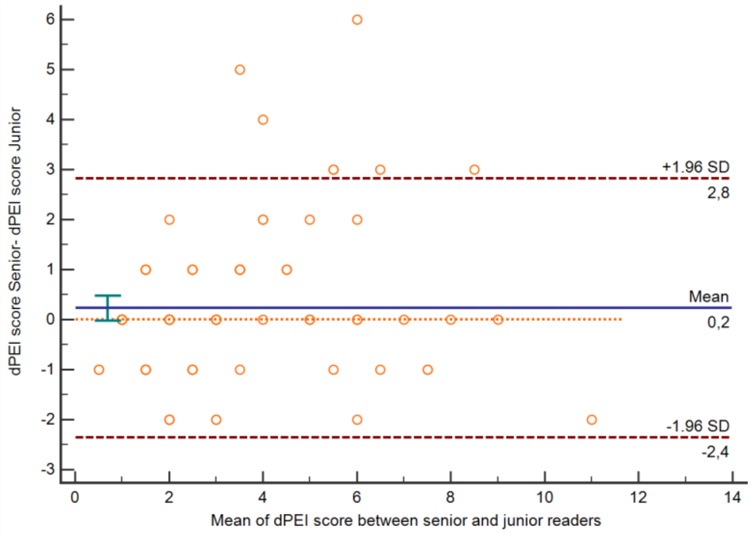

© 2023 Thomassin-Naggara I et al. *JAMA Network Open.*
