# Supplementary material for: Multicenter External Validation of the Deep Pelvic Endometriosis Index Magnetic Resonance Imaging Score
Source: JAMA Netw Open. 2023 May 4;6(5):e2311686. doi: 10.1001/jamanetworkopen.2023.11686 (PMC10160872; doi:10.1001/jamanetworkopen.2023.11686)
Supplement: Supplement 2. — Data Sharing Statement [file jamanetwopen-e2311686-s002.pdf]

Data Sharing Statement

Thomassin-Naggara. Multicenter External Validation of the Deep Pelvic Endometriosis Index Magnetic Resonance Imaging Score. *JAMA Netw Open*. Published May 04, 2023. doi:10.1001/jamanetworkopen.2023.11686

Data
Data available: No
